# “Ambivalence Perception” the Consequence of Exposure to Pregnancy in Iranian Adolescent Women: A Qualitative Study

**Published:** 2018

**Authors:** Maryam Moridi, Farkhondeh Aminshokravi

**Affiliations:** - Department of Health Education and Health Promotion, Faculty of Medical Sciences, Tarbiat Modares University, Tehran, Iran

**Keywords:** Adolescent, Pregnancy, Women’s health

## Abstract

**Background:**

Adolescent pregnancy is an important health problem, significantly related to negative effects on the health of both adolescent mothers and their babies. Little is known about adolescent pregnancy from the perspective of the adolescents, especially in developing countries. The present study aimed to explore the perception of response to pregnancy in Iranian adolescent women.

**Methods:**

This conventional content analysis was conducted from November 2015 to October 2016 in Guilan Province (In the north of Iran). Data were collected through unstructured interview with 24 married women aged between 14 and 18 years old. The participants were recruited using a purposive sampling method. Interviews began with a general question and were followed with some probing questions, and were continued till data saturation was reached.

**Results:**

“Ambivalence perception” was the main theme that merged in this study. Two other categories comprised the content of interviews: “Improving positive effects of pregnancy” and, “Diminishing negative effects of pregnancy” which were merged from nine sub-categories.

**Conclusion:**

The experiences of pregnancy were not completely undesired and negative as the teenage mothers expressed a feeling of satisfaction with the birth of their children. This finding will help health educators to develop cultural sensitive programs, activities, and educational interventions that assist adolescent mothers to deal with this ambivalent perception of pregnancy.

## Introduction

Adolescent pregnancy is a major health problem in the 21st century (1) and it is considered as the first killer in girls aged 15–19 years old (2). Every year, approximately 16 million and one million births occur in girls aged 15–19 and under 15 years old respectively, of which, 95% occur in low and middle income countries (3). Universally, adolescent pregnancy has been identified as a negative event because it is related to significant adverse effects on individual, social, economic, and health outcomes. Also, pregnancy in adolescence negatively effects employment and educational opportunities (4). Babies born to adolescent mothers are more likely to die suddenly than those that are born to adult mothers due to low birth weight and prematurity (5).

Iran’s population of adolescents is high, and the age specific fertility rate in this group has been raised from 25 to 35 *per* 1000 adolescents from 2010 to 2011 (6). Iran’s population policy has shifted from a decrease to an increase in the subsequent decades. Therefore, it is predicted that the fertility rate of Iranian adolescents will be elevated by 2025 (7). However, there are differences in adolescent fertility rate across the country–with Sistan-Baluchestan and Guilan having higher and lower rates of adolescent pregnancy, respectively.

It is noteworthy to mention that, based on the religious and cultural context of Iran, women give birth following their marriage, so they are confronted to simultaneous events such as marriage, pregnancy, and mothering in transition to adulthood. Among Iranian women experienced teenage pregnancy in their reproductive life, most studies have shown that adolescent pregnancy not only has significant negative effects on adolescent women and their families, but it is also a significant challenge for public care system in attaining the Millennium Development Goals. However, some studies recently showed that pregnancy has some positive effects on the life of adolescent pregnant women (8). Perceived benefits to adolescent pregnancy include developing relations with parents and family, happiness, performing gender roles (9), feelings of responsibility, maturity (8), independency (10), and stabilization of self-esteem (11).

During the last 20–25 years, the existing data on adolescent pregnancy are often based on quantitative studies. The researches usually address the physiological results of pregnancy during this period, the risks of pregnancy, and ways to control them. So, what adolescents experience during the period has been studied to a lesser extent (12). Further, recognizing the phenomenon of pregnancy in adolescents by using qualitative approaches can lead to better insight of this phenomenon from the perspective of adolescents and can facilitate the promotion of knowledge of those who are responsible for providing services to adolescents (10). Also, the experience of pregnancy in adolescence is a phenomenon that is strongly influenced by cultural, social, political, and religious contexts of every community. Since the dominant focus of universal studies is on the experiences of pregnancy in adolescence outside marital relationship, this study pictured a new and different experience of this phenomenon within marital relationship. These experiences can be good directions in designing health promotion programs and developing mental and physical health promotion strategies tailored to the needs of adolescents. Considering the importance of the matter and conflicting perceptions about adolescent pregnancy, this study with the aim of exploring the perception of response to pregnancy in Iranian adolescent women was designed.

## Methods

### Study design:

This study was part of a doctoral dissertation, which used qualitative content analysis to explore the experiences of Iranian married adolescent women in confronting pregnancy. Content analysis is a systematic approach that provides a new insight into a particular phenomenon. It leads to valid deduction from data and is appropriate for exploring the experiences and views of people towards the issue of interest (13). The current study was conducted from November 2015 to October 2016 in Guilan Province (North of Iran).

### Participants and setting:

In this study, participants were pregnant women from the beginning of pregnancy till one week after the delivery, who were chosen from urban and rural primary health care centers (PHCs). The PHCs were selected randomly from several towns and villages of Guilan province. At first, teenage women who had inclusion criteria and had been registered in the antenatal clinics of PHCs and/or a referral obstetric hospital in Rasht city (The capital of Guilan province) were recruited applying purposeful sampling method. For this purpose, the telephone numbers of potential participants were selected from registry lists. Next, the first author contacted the potential participants and, the study information was provided to them. Once the pregnant adolescent women agreed to participate, the place, time and date of the interview were determined as they desired Only married women, aged 14–18 years, living in Guilan province, speaking, and understanding Persian language, willing to participate in the study, and able to convey their experiences were included in the study. The congenital malformation in fetuses or medical and obstetrical problems in adolescent women were considered as exclusion criteria.

### Data collection and analysis:

The main technique of data collection was the interview, which was conducted by the first researcher who was supervised by two other experts. The data collection was performed by unstructured, indepth and face to face interviews with 24 participants. Duration of the interview sessions varied from 30 to 90 *min*.

At the beginning of the interview, the participants were asked a general question: “Would you please explain your experiences of pregnancy?” or “Could you please tell me how you felt when you were informed of the pregnancy?” Then, when necessary, some probing questions were asked (*e.g*. “What do you mean exactly?” or “Would you please explain more?”) to obtain richer and deeper information. All interviews were audio-recorded in mp3 format and transcribed verbatim in Persian language prior to the next interview. Concurrent with data collection, data analysis was done using Graneheim and Lundman’s method including transcription of the whole interview immediately after each interview, reading the entire transcription of the interview to achieve an overall understanding of its content, specifying semantic units and basic codes, classifying initial similar codes in more comprehensive categories, and ultimately, extracting more abstract themes from the categories (13) (Table 1). Data saturation was obtained after interviewing with 24 participants and when no new concepts or categories were developed thereafter.

**Table 1.** Categories and sub-categories of ambivalence perception

**Theme**	**Category**	**Sub-category**
**Ambivalent perception**	Diminishing effects of pregnancy	Social isolation
		Unable to perform mothering roles
		Fear of pregnancy outcomes
		Fear of stigmatization
		Fear of delivery
	Improving effects of pregnancy	Feeling of independence and confidence
		Improvement in marital life
		Fundamental change in adolescent women’s life
		Eliminating the loneliness

### Trustworthiness:

The following strategies were used to ensure trustworthiness in this study. The following strategies were used to enhance credibility. First, according to the study question, a conventional content analysis as an appropriate research methodology was done with the careful supervision of the research team. Second, audio-records and transcripts, multiple data sources, including field notes, observation, and diaries were used. Third, the interviewer had a prolonged engagement with data. Finally, all interviews were carried out with an interviewer trained in conducting qualitative research and interviewing techniques. For raising the study confirmability, at the end of the study, the methods of member checking and peer check were used. Researchers tried to enhance transferability by choosing participants with maximum variation. In addressing the dependability, the clarify codes were used, and the method of study and data gathering were explained in details; in addition, all the categories were written based on participants’ quotations.

### Ethical consideration:

This study was approved by the Ethics Committee of the Research Deputy at Tarbiat Modares University as well as the Ethics Committees of the Research Deputy and Health Deputy of Guilan University of Medical Sciences. Also, each participant and her spouse or her family members signed the written informed consent before the interview. It must be noted that this article is part of a larger qualitative study and the article presents a part of study findings.

## Results

### Demographic characteristics:

The participants were twenty-four married women aged between 14 and 18. Most of them were primigravida, housewife, and had 8 years of education. The characteristics of participants are reported in table 2.

**Table 2.** Charestrictics of the participants

**Participant’s code**	**Age**	**Gestational age**	**Gravidity**	**Parity**	**Duration of marriage**	**Educational level**	**Job**	**Husband’s age**	**Husband’s educational level**	**Husband’s job**
**1**	17 yr	One day after birth	1	0	2 yr	8 yr	HW	32 yr	11yr	SE
**2**	16 yr	28 wk, 4d	1	0	4 *mn*	11 yr	HW	31 yr	11yr	SE
**3**	15 yr	One day after birth	1	0	2yr	10 yr	HW	24 yr	11yr	SE
**4**	16 yr	Two days after birth	1	0	1 yr	6 yr	HW	21 yr	11 yr	SE
**5**	17 yr	One day after birth	1	0	2 yr	9 yr	HW	24 yr	7 yr	SE
**6**	14 yr	One day after birth	1	0	1 yr	6 yr	HW	24 yr	11 yr	SE
**7**	17 yr	37 wk	1	0	10 *mn*	5 yr	HW	20 yr	14 yr	US
**8**	17 yr	39 wk, 2d	1	0	1 yr	6 yr	HW	26 yr	9 yr	SE
**9**	17 yr	38 wk. 1d	2	0	4 yr	5 yr	HW	24 yr	11 yr	SE
**10**	15 yr	27 wk, 2d	1	0	10 *mn*	8 yr	HW	22 yr	11 yr	SE
**11**	16 yr	16w, 5d	1	0	2 yr	8 yr	HW	25 yr	10 yr	SE
**12**	18 yr	14 wk	1	0	7 *mn*	8 yr	HW	19 yr	11 yr	SE
**13**	18 yr	18 wk	1	0	2 yr	8 yr	HW	21 yr	5 yr	SE
**14**	17 yr	29 wk	2	1	4 yr	10 yr	HW	23 yr	11 yr	SE
**15**	16 yr	36, 4d	1	0	1 yr	5 yr	HW	19 yr	6 yr	SE
**16**	16 yr	32 wk, 3 d	2	1	3 yr	7 yr	HW	22 yr	8 yr	SE
**17**	14 yr	22 wk	1	0	1 yr	9 yr	HW	24 yr	16 yr	SE
**18**	17 yr	13 wk, 2d	1	0	1 yr	9 yr	HW	22 yr	8 yr	SE
**19**	17 yr	19 wk, 4d	1	0	1 yr	7 yr	HW	20 yr	8 yr	SE
**20**	16 yr	35wk, 6d	1	0	2 yr	8 yr	HW	27 yr	16 yr	SE
**21**	18 yr	31wk, 2d	1	0	1 yr	7 yr	HW	25 yr	13 yr	US
**22**	18 yr	26 wk, 3d	2	0	3 yr	6 yr	HW	22 yr	9 yr	SE
**23**	17 yr	8 wk	1	0	6 *mn*	10 yr	HW	24 yr	7 yr	SE
**24**	14 yr	21 wk	1	0	1 yr	6 yr	HW	28 yr	11 yr	SE

wk: weeks; d: days; yr: years; *mn*: month; HW: SE: Housewife; Self-Employed; US: University Student

### Ambivalent perception:

Ambivalent perception of pregnancy was the main theme emerged from the participants’ prospective as a result of exposure to pregnancy in adolescent women. Exposure to pregnancy was symbolized as a condition leading to improving or diminishing the adolescents’ lives. This theme consisted of two categories which pointed to the conditions that adolescent women had perceived in their daily lives following exposure to pregnancy. These categories incorporated 9 sub-categories shown in table 1.

### Diminishing effects of pregnancy:

This category articulated that the adolescent women perceived that pregnancy would diminish their future life. The diminishing effects of pregnancy consist of 5 sub-categories described in the following.

### Social isolation:

Social isolation refers to the feelings which the participants supposed to be excluded from their life because children disturb their social relationships. The participants who had another child believed the concurrent troubles of two children would diminish their social relationships. The following excerpt is taken from one of the participants’ interviews. *“Wherever I want to go somewhere such as a market or a party, I have to carry two babies alone and it is cumbersome for me…, Therefore, I prefer to stay at home”* (P14).

Some participants pointed out that after pregnancy the attention of their husband declined because they were reluctant to accept the pregnancy. A participant said *“I feel that my spouse is rejecting me because he doesn’t want the child”* (P20).

### Unable to perform mothering roles:

This sub-category refers to the feelings which the participants pointed out they were not physically and psychologically ready to become pregnant because they did not have enough experience. The participants felt that they are so young to be experiencing motherhood, thus they had anxiety and were concerned about how they would manage their mothering roles and responsibilities. These women had different reasons for being unable to perform mothering roles. For instance, some participants presumed that the responsibilities of mothering are a heavy duty and it was more trouble with the lack of their spouse or family’s support. *“I do not know anything about how to take care of a baby, my mother completely looked after my first child, but now she is dead and I do not have any support”* (P14). Some teenage women underestimated their ability in performing mothering roles. *“When I heard I am pregnant, I was disappointed and scared, it is a big responsibility, I’m still a child, I cannot look after a baby”* (P13).

### Fear of pregnancy outcomes:

This sub-category refers to participants’ apprehensions about the physical change during pregnancy and adolescent women believed that pregnancy would have high risks for young mothers. A participant expressed *“I had a lot of fears about pregnancy changes…, my abdomen will be bigger, it is very small, and how will it be so big?”* (P19) and, another woman insisted *“I want to terminate it, I heard that pregnancy in young ages will be dangerous for mother…, my cousin is a midwife, she said to me it is possible that my baby may be born prematurely”* (P8).

### Fear of stigmatization:

This sub-category refers to the participants’ apprehensions about other people’s opinion on their early pregnancy. Some participants wanted to terminate or hide their pregnancies from their spouse, family, friends and others due to their disagreement, blame, and mockery for the early pregnancy. One participant said: *“My motherin-law insisted that I abort my baby…, she fears about people’s talking about our unplanned pregnancy”* (P6). Another participant added: *“I said to my spouse that it is shyness…, I want to abort it due to fear of what people will say…, for example, they will say: she bore a child immediately after getting married, maybe she was pregnant before it”* (P8).

### Fear of delivery:

One of the major concerns expressed repeatedly by participants both before and after delivery was the fear of delivery, especially natural vaginal delivery. Some participants fear the pain of delivery, for instance, one woman expressed: *“After a few months passed, I begin to be really afraid of delivery, the only fear that I have, is fear of delivery, the rest is all happiness”* (P7). One participant said: *“Natural delivery has pain, but in cesarean I do not feel any pain because I will be unconscious”* (P20).

### Improving effects of pregnancy:

In this category, participants believed that pregnancy has improved their lives and made them more independent, confident, brought about a better marital life, a fundamental change in their life and eliminated their loneliness. This category consisted of four sub-categories described as follows:

### Feeling of independence and confidence:

This sub-category refers to the participant’s tendency to be independent which was achieved after becoming pregnant and they perceived that pregnancy led to feelings of ownership of a child, self-esteem, being proud of their reproductive ability, proved their ability of nurturing and looking after a child alone. One of the participants said, *“Now, I’m really a mother, I felt proud, I am nurturing a person in my body and I am going to add a person to the community”* (P7). Another participant said, *“Always I try to be independent from my family and now, I feel I have become more independent”* (P19). A woman interviewed two days after delivery expressed that *“I think I must be an independent woman, I want to do everything for my child by myself, I do not need anyone’s help, I want to have a job and spend money for my child”* (p1).

### Improvement in marital life:

Some participants believed that after becoming pregnant, their relationship with their spouse was closer and he expressed more love to them, and pregnancy caused a change in their spouse’s behavior, eliminated the conflicts, and they became a fortunate couple and have a sweeter life. In this regard, one participant said, *“My spouse is ten times closer to me.., my spouse does anything that I want at any time…, all couples sometimes have conflicts, now, that little conflict has disappeared because of pregnancy. We have become a lucky couple”* (P7). Another participant said, *“After pregnancy, my spouse changed, his kindness increased, I feel he loves me more”* (P8).

### Fundamental change in adolescent women’s life:

This sub-category refers to participants’ perception towards pregnancy as a new life. Participants experienced inward and outward fundamental change in their lives. In the outward change, participants symbolized pregnancy as a new condition leading to change in their body appearance, and lifestyle change. They began to think of themselves as mothers and to accept mothering responsibilities. A participant exemplified this fundamental change as follows *“I feel my life has completely changed, for example, how the life of a single person is different from a married person, when a person marries, her life completely changes, pregnancy is like that, a pregnant woman experiences different things, now, I think a few months later I will give birth to a baby, I should take care and nurture a baby, I always think about these issues that are different from before”* (P10). Another participant said, *“My daily habits changed due to pregnancy, now I must pay attention to some things…, for example, I am a tailor, but now I cannot do it because of the pregnancy…, I was a person who went to market every week but now, they told me, you must rest at home”* (P20). The inward fundamental change in women’s life was stated as enhancing enthusiasm and hope for my life. *“It seems I have more enthusiasm for life, for example, I wear a good dress every day, my spouse likes me more, I eat ice cream every day for my child, so that my child will become sweeter”* (P15).

### Eliminating the loneliness:

Some participants perceived that becoming pregnant and having a child could remove their loneliness. This quotation was especially stated by adolescent women who were alone at home for a long time every day and women who have large age disparities with their spouses. *“I’m usually alone at home…, I hope the baby will be a girl, because girls are closer to their mothers.., I always think that when she grows up, we will go everywhere together,… I will play with her, and my house will be full of sound”* (P5). Another participant said *“My spouse is much older than me and often we are apart for several days, I want to remove my loneliness with a baby, I’d like to have a friendly relationship with him”* (P1).

## Discussion

Adolescent women exposed to pregnancy experienced the spectrum of perceptions that had continuum range from the improvement to diminishing of their future life. The adolescent mothers expressed different degrees of positive or negative perceptions about their pregnancy. The positive perceptions in the adolescents with higher perceived support and acceptance from family, especially their own mothers and spouses and better readiness for pregnancy, were predominant and they interpreted that pregnancy would lead them to becoming more responsible, empowerment, independence, enthusiasm, appreciation and promotion of their marital lives. However, in these women, negative perceptions about the possible complications of future life for herself and the child existed too, whereas in the adolescent women with less support and acceptance, and less readiness for becoming a mother, higher degrees of negative perception and lower degrees of positive perception were demonstrated. Also, the couples who had planned pregnancy had better acceptance and more positive feeling regarding pregnancy and childbearing. These results were similar to a qualitative study from Karaj, which showed that adolescent women who had consensual marriage or preplanned pregnancy expressed good feelings about pregnancy and having a baby. But, the participants who had got married non-consensually or became pregnant in an unplanned way had bad feelings about the child (12).

One of the causes of conflict and existential ambivalence is that adolescent parenthood goes against the plans or objectives that society has for individuals, such as school performance, professional preparation, obtaining a suitable job, the establishment of a stable, lasting and loving relationship and, only then, reproduction within the relations of matrimony. When life situations do not occur more or less within this defined order, it can result in problems for adolescents (14). Also, this opposite feeling is a natural event that is also characteristic of other interpersonal relationships, given that the complexity of human relationships permits the co-existence of the most diverse types of feelings. On the other hand, adolescence is a transitional stage and preparation for crossing childhood and entering into adulthood (15). During this period, some important experiences are acquired in the process of growth and development. In addition to the experiences of physical and sexual maturation, adolescents experience the movement towards economic and social independence, identity development, acquisition of skills needed to communicate and the acquisition of adult roles and capacities for reasoning and abstract thinking (Word Health Organization, 2016) (16) and they encounter important and diverse developmental challenges. The beginning of pregnancy is also a significant transition period during which women experience several multilateral changes in their life. According to psychologists, pregnancy is a critical period in terms of social and psychological stresses that reflects the crisis of maternal identity formation (17). Taking mother’s role during adolescence means a teenage girl faces with parental responsibility since she has to cope with developmental tasks of maturity such as shaping her identity and the onset of sexual relations. Therefore, teenagers have less opportunity to cope with the physical, emotional and psychological changes of adolescence before facing with the changes and challenges of pregnancy (11). Many studies have introduced the lack of preparation to get pregnant as the main problem of pregnant teenagers. Thus, many teens tend to delay pregnancy until they become ready and gain the necessary competencies (10, 18, 19).

The ambivalence perception of pregnancy is consistent with several previous studies (7, 14, 20). According to Carvalho (2010), the opposite feeling about pregnancy is not completely absent. The experience of pregnancy is related to ambivalence emotional perception (14).

Generally, based on the perceived social and familial support and women’s willingness to become pregnant, the ambivalent feeling varied from positive to negative. Some young mothers were very positive about their experiences of motherhood and they felt it had been worth the privilege of having children. They described having a child as changing their life and allowing them to grow up. The women were proud of their children and wanted the best for them and in some participants, they wanted to prepare everything for them in a way that they themselves did not have access to. They were also realistic about their responsibilities; some said being a full time mother is important while the children are very young, but this did not mean that they did not have plans for the future. Some participants had decided to return to work or education with the assistance of their families to look after their children, while some participants were very negative about their experiences of motherhood and they have many concerns such as fears of stigmatization, pregnancy outcome, delivery, inability to perform mothering roles and social isolation.

The results of present study indicated that, more than half of the participants have positive feelings in exposure to pregnancy. Regarding the main reason for this finding, it is worth noting that pregnancies occur within the marital relationship in concordance with Iranian community. In addition, based on the religious and cultural context of Iran, pregnancy and childbearing are holy and valuable events. So, the families provide a supportive environment for pregnant women which resulted in coping well with pregnancy’s stresses and challenges. This finding is supported by a considerable number of studies; for example, the study conducted by Higginbotto et al. (2006), and McMichael (2013) demonstrated that adolescent pregnancy should not be viewed only through a risk prevention lens that emphasizes the negative consequences for adolescent mothers and their babies (21, 22). Some studies indicated that there is an associated sense of maturity and responsibility (23). Many young mothers felt they were stronger and more competent (24).

In this study, adolescent pregnancy brought women closer to their spouse and improved their marital relationship and they valued having a child whom they loved and who loved them back. This finding is similar to other studies of adolescent pregnancy and parenthood (23–25). Some studies have reported that pregnancy and motherhood can strengthen relationships and feature the women’s place in a relationship, marriage and within the community (7, 25–27).

In addition, pregnancy was perceived as inward and outward fundamental changes for the participants. Inward changes indicate an internal feeling of enthusiasm due to becoming pregnant and, pregnancy caused the participants to feel they were competent enough to bring up a fetus in their own body. Outward change was accompanied with a sense of stability, identity, purpose and responsibility. For them, pregnancy was the main reason that family members, relatives and friends referred to them as a mother; so, these findings are in agreement with several studies (7, 23, 25, 27, 28).

Despite these positive experiences, adolescent mothers face several challenges. Fear of natural delivery and women’s tendency toward cesarean section was one of the main concerns of participants. According to a study from Iran, the selection of a birth method is influenced by multiple complex factors such as cultural values, beliefs and available psychosocial support and misconceptions regarding cesarean section. In Iran, women’s tendency toward cesarean section is increased because of birth medicalization as well as socio-cultural and economic norms (29).

This study indicated that adolescent women have problems with holding friendships and social activities with former friends and family members who cannot understand the stresses of adolescents, and new lifestyle as a mother. Consequently, adolescent mothers may feel alone and abandoned by their friends and family. Also, they may diminish their relationships due to fear of stigmatization. A study showed that adolescent mothers are stigmatized by the community and are likely to experience violence and rejection from spouses (30). Adolescent mothers need support from both their family and friends and being part of a group creates a sense of acceptance, socialization, and stability. Therefore, they require further opportunities to find new friendships with other adolescent mothers who share their experience of being a parent and provide an important source of peer support and reassurance.

The results of the present study are similar to the qualitative studies from other cities of Iran. Mohammadi et al. (2016) in a phenomenological study from Ahvaz showed that adolescent pregnant women experienced fast development because they responded to concurrent occurrence of new and important life events such as transition to adulthood, getting married, becoming pregnant and being a mother without any readiness for coping with these roles. Fast development was merged from three themes. The first theme was unexpected development which demonstrated that most adolescent women were not ready physically, psychologically, emotionally and economically to become pregnant; also, some of them believed that pregnancy was imposed to them and some of them fear of pregnancy outcomes and mothering roles. The second and third themes showed that adolescent women experienced a new life, intensified egocentric perception and dual self-perception (7). Another study from Karaj showed that adolescent women react to pregnancy physically, psychologically and spiritually. Psychological reaction to pregnancy included feeling, concerns and fears about pregnancy, spouse, and child. The participants feelings affected by marital status and becoming pregnant in preplanned way and their concerns included worry about labor, premature childbirth, baby’s health, its condition, and abortion, and fears were consisted of being afraid of pregnancy due to young age, being afraid of childbirth, fearing people’s jinx because of pregnancy at young age, being concerned about how to raise the child alone, being worried of not having menstruations, being afraid of what the doctors would say about baby’s health, fearing that the baby may be strangled to death, and fear of harming the child by lying on their side (12).

Adults and adolescent have many different psychological traits such as harm avoidance, lower level of behavior control and increased risk taking and the lack pertaining to tradition, values and morals (31). These differences may affect their perception of exposure to pregnancy so, a feeling of ambivalence is the result of adolescent exposure to pregnancy, which can be different in adults. It is suggested that this study be performed on adult pregnant women to identify the probable differences.

The main limitation of this study was that the participants were selected from among women visiting PHCs or the selected hospitals, indicating that voices from those women who did not have antenatal care visits or chose not to participate are not represented. Another limitation of this study was that the differentiation between planned and unplanned and wanted or unwanted adolescent pregnancies was not considered in inclusion criteria. Although this research provides data for understanding the experiences of adolescent women about their perception of pregnancy, it is a qualitative study with potential biases resulting from selection issues, so its findings are not representative and therefore not generalizable to all adolescent pregnant women. In addition, qualitative data could be subject to multiple interpretations.

## Conclusion

Iranian adolescent women experienced an ambivalent perception in exposure to pregnancy, which ranged from positive to negative perceptions. For some participants, pregnancy reinforced their identity and self-perception but other participants were involved in challenging perceptions. The study results have implications for policymakers and health care providers to design and develop health programs and culturally sensitive intervention tailored to the needs of pregnant women. Ambivalence perception concept offers an explicit understanding to design comprehensive services based on challenges from exposure to pregnancy. According to the results of this study, one of the predictors of the levels of these dual perceptions was the perceived support from family, society and the healthcare system. Therefore, it is necessary that families, communities, and health care systems provide comprehensive support to adolescent women for improving negative feelings toward positive feelings in exposure to pregnancy, which could have positive effects on the quality of life of young mothers and their babies. Also, applying the results of the present study may develop adolescent friendly maternal care services. Moreover, these data indicated that care given to adolescent mothers should take into consideration not only the theoretical and chronological aspects, but also the psychosocial and cultural factors related to parenthood in this social group (32). The study results have implications for policy makers and health care providers to design health promotion programs and developing mental and physical health promotion strategies tailored to the needs of adolescents. Further research, particularly on the long-term effects of teenage motherhood is suggested.
